# Muscling in on immunity: the role of muscle in the insect immune response, demonstrating the value of a whole organism perspective

**DOI:** 10.3389/fimmu.2026.1749942

**Published:** 2026-02-25

**Authors:** Shelley A. Adamo

**Affiliations:** Dept. Psychology and Neuroscience, Dalhousie University, Halifax, NS, Canada

**Keywords:** adaptive immunopathology, ecoimmunology, immunometabolism, insect immunology, physiological network, trade-offs

## Abstract

Insects have robust immune systems to protect themselves against pathogens. However, the immune system relies on the support of a number of organs for effective immune defense. Muscle plays an unexpectedly key role in both cell-mediated and humoral immunity in insects. During an immune response, muscle responds to increasing cytokine levels by producing antimicrobial peptides. Muscle also shifts resources toward the immune system by releasing myokinins and other factors. These compounds reduce insulin-like peptide release from the brain. In addition, muscle undergoes insulin resistance, further reducing its uptake of glucose. Muscle also donates its own resources, breaking down glycogen to support the hemocytes’ need for glucose during cell-mediated immunity. However, this support of immune function results in a decline in muscular capacity, leading to reduced anti-predator behavior and increased predation. This physiological trade-off between muscle and immunity may help explain why sick animals typically reduce exposure to predators by increasing shelter use. In addition, muscle’s ability to regulate the flow of resources in the body suggests that it may also play a role in mediating trade-offs between immune function and other life history traits, such as reproduction. Muscle should be considered as a research target by ecoimmunologists. Some immune-mediated effects, such as insulin resistance and chronic inflammation, may have adaptive functions when viewed from the perspective of fitness maximization under adverse conditions.

## 1 Introduction

Infection is a potentially lethal event in the life of an organism. Over time, animals have evolved specialized cells and organs (i.e. the immune system) to destroy pathogens. However, defending against pathogens is expensive, and the magnitude of the response waxes and wanes depending on a number of factors (1, 2). Moreover, pathogens are patchily distributed in the environment. The variability in the immune system’s resource requirements favors the evolution of a distributed immune network; one that taps into other organ systems when necessary. This type of organization reduces the amount of resources tied up in the immune system when not required.

Evidence for the cross-organ reach of the immune system can be found in the physiological trade-offs between the immune system and other life-history traits (e.g. in insects: 3–5). Immune system activation can result in organ damage, leading to decreased lifespan (6). Animals negotiate the balance between immune defense, the needs of other physiological systems, and avoiding immunopathology, by reconfiguring physiological networks, thereby optimizing fitness (7). Therefore, understanding how immune systems operate requires a whole network perspective, incorporating the contributions of other organ systems (e.g. 8, 77, 7, 9–12). This review discusses the role of skeletal (i.e. somatic) muscle in immune defense in insects.

## 2 Brief summary of insect immunology from a network perspective

Insects have robust immune systems, allowing them to flourish in pathogen-laden environments. Insect immune systems have two principle components: cell-mediated immunity and humoral immunity (13, 14). Cell-mediated immunity consists of the immune cells of the blood (i.e. hemocytes). These cells can destroy invading organisms using a variety of methods including phagocytosis, encapsulation and nodulation (13, 14). Humoral immunity includes the proteins and peptides found in hemolymph that can destroy invading organisms (15). One of the most important components of humoral immunity is the phenoloxidase pathway (16). Once activated, this pathway generates reactive molecules (e.g. reactive oxygen species, ROS) and melanin that kill invaders (17). Antimicrobial proteins and peptides also destroy pathogens, and their production is induced by pathogen presence (15). They are made by the fat body (an immune and energy storage organ, (15) and hemocytes (e.g. 18).

The immune system consumes substantial resources in insects (e.g. 19, 20). In *Drosophila*, activating cell-mediated immunity requires shunting large amounts of glucose to hemocytes (9). Activated hemocytes shift their metabolism towards glycolysis (i.e. the Warburg effect), allowing them to generate ATP quickly; however, this shift also requires large amounts of glucose (9). During an immune response, hemocytes consume 27% of the animal’s total glucose budget (20). Hemocytes also require increased amounts of amino acids, such as glutamine and methionine, to allow them to alter their cytoskeleton and encapsulate invaders (9, 17). These changes in hemocyte metabolism allow for a rapid and effective response (21), that is critical for overcoming a pathogen (22). Humoral immunity is costly too. The phenoloxidase pathway uses tyrosine-derived compounds as a substrate (17, 23). It also requires an increase in the use of antioxidants (e.g. glutathione) to protect the host from immunopathology (24). The recruitment of resources by the immune system results in a decline in resource availability for other systems. For example, activation of immune responses results in slower growth and development in the caterpillar *Manduca sexta* (25) and reduces reproduction in a range of insect species (4). However, without these additional resources (e.g. glucose), disease resistance declines, as shown in *Drosophila* (e.g. 21, 26).

**Figure 1:**
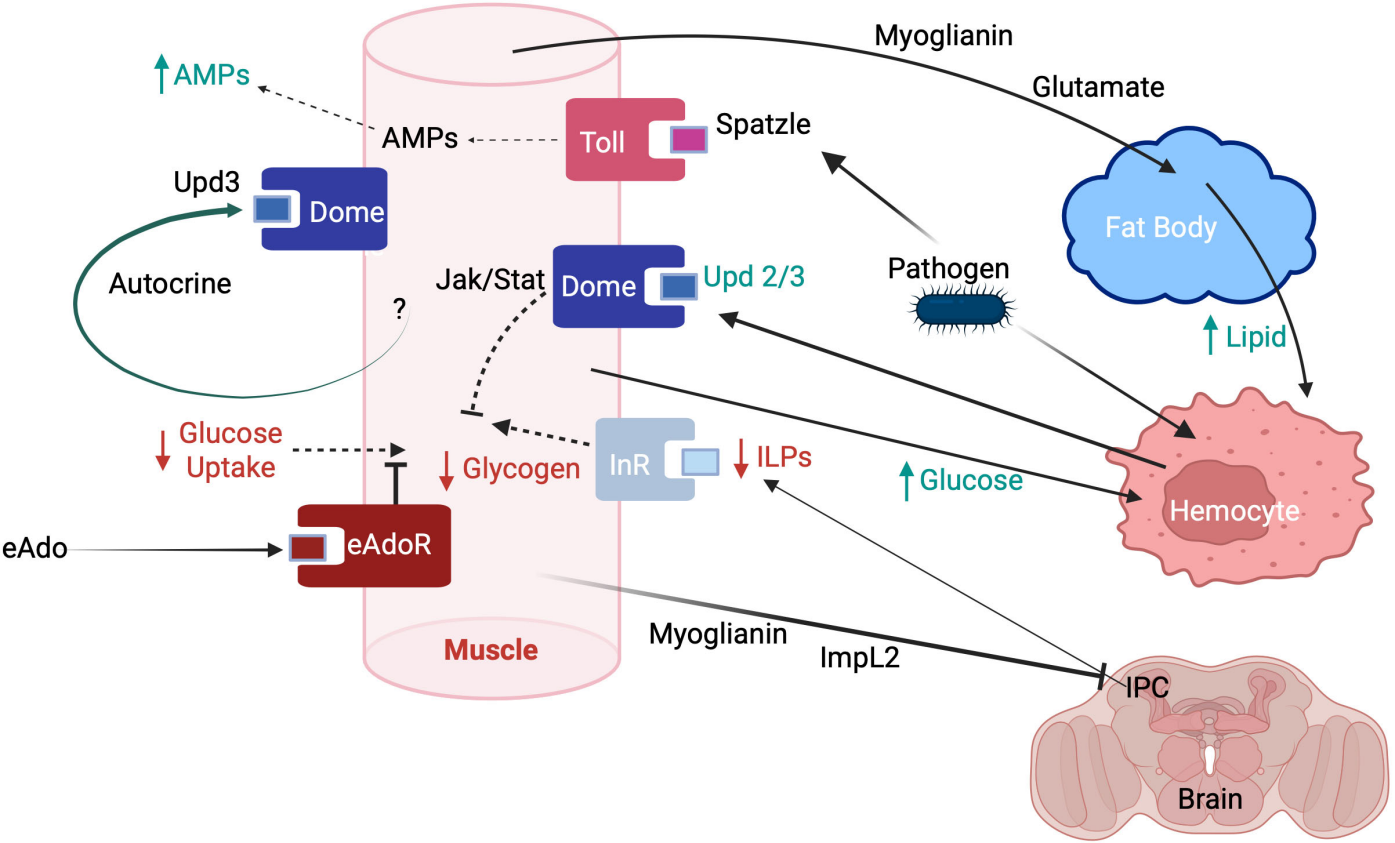
The immune system and muscle are interconnected. Muscle has receptors for cytokines (e.g. Toll and Dome receptors), allowing it to receive signals from hemocytes (e.g. cytokines Upd2 and Upd3) and the humoral immune system (e.g. Spatzle). In response to the cytokine Spatzle, muscle produces AMPs. Activation of the Jak/Stat pathway by Upd3 leads to reductions in glycogen synthesis and glucose uptake in muscle, probably via Jak/Stat interactions with the insulin signaling pathway. During an immune response, muscle releases glutamate, and myoglianin, inducing the fat body to release lipid. Muscle also releases ImpL2 and myokinins that result in reduced ILP (insulin-like peptides) release from insulin producing cells (IPC) in the insect brain. Green text represents compounds that are increased during an immune response; red text represents compounds that are decreased. InR: insulin-like peptide receptor, eAdo: extracellular adenosine. Adapted from: Yu et al. (12)McMullen et al. (26)Alfa and Kim (32)Droujinine and Perrimon (33)Bajgar et al. (34)Zhao and Karpac (35). Created in https://BioRender.com.

Immune systems in insects are deeply embedded within dense intra- and inter-organ signaling networks (7, 12, 27–31) (**Figure 1**). The immune system uses chemical signals (i.e. cytokines) to help coordinate its own response (13, 36). For example, plasmatocyte spreading peptide is vital for hemocyte function in *M. sexta* (37). Cytokines can act individually, synergistically or antagonistically (13). Many organs have receptors for cytokines, including the central nervous system (CNS) and muscle (36). The existence of these cytokine receptors reflects, in part, the participation of cytokines in multiple physiological functions in insects (e.g. immunity and development, 36). For example, interconnections between the immune system and muscle occur even in the absence of an immune response. In *Drosophila*, some hemocytes reside in close proximity to skeletal muscle, and these hemocytes chronically release low levels of the cytokines Upd2 and Upd3 (38). These cytokines bind with Dome receptors on muscle, producing a baseline level of activity of the Jak/Stat pathway (38). If muscles lack the receptor for Upd2 and Upd3 (i.e. Dome), muscles develop lipid inclusions, and the climbing ability of the fly declines (38). This immune-muscle connection demonstrates that the immune system also helps regulate muscle metabolism. Cytokine receptors in organs such as muscle are also activated during an immune challenge (12). During an infection, hemocytes increase their cytokine release, raising activity in the Jak/Stat pathway in muscle above basal levels (38). Therefore, cytokine receptors provide a mechanism by which a variety of organs can participate in an immune response.

Additionally, many cells of the immune system contain receptors for signaling molecules from ‘non-immune’ organs, such as the endocrine system (i.e. hormones, 30) and CNS (e.g. 39). The ability of the immune system to respond to signals from other organs enables it to alter its responses depending on internal and external conditions. For example, resource availability can modulate immune responses via the insulin signaling pathway, as shown in *Drosophila* (12, 40). Olfactory cues indicating the presence of pathogens can increase immune responsiveness via signals from the CNS (39). It is these inter-organ connections that allow the immune system to gain access to resources, to recruit other organs to assist, to respond to competing needs within the organism, and to respond to pathogen prevalence in the environment. These connections allow insects to have an immune response of a type and magnitude that enhances its fitness. Animals are selected to maximize fitness, not immune function.

## 3 The role of skeletal muscle in immune function

Muscle plays a crucial role in immune defense in both mammals (41) and insects (42–44). One of the first indications that muscle plays a direct role in the immune response in insects was the discovery that *Drosophila* muscle produces antimicrobial peptides (AMPs) (e.g. drosocin, drosomycin and diptericin) in response to infection (42). Moreover, these AMPs are important for the successful clearance of bacteria (42). In addition to AMPs, muscle can also produce pathogen recognition molecules (e.g. *Antheraea pernyi* (Lepidopteran) 44), further contributing to humoral immunity. During an immune challenge, muscle also upregulates the expression of cytokine receptors such as Toll and Dome in *M. sexta* (45), potentially increasing muscle’s sensitivity to immune signals when pathogens are present. Therefore, muscle acts as an auxiliary immune organ, responding to cytokines with the production of key immune proteins. By recruiting additional tissues like muscle, the immune system maximizes its response to life-threatening infections.

In addition to playing a direct role in immunity, muscle also plays a critical role in mediating the metabolic changes needed for a successful immune response (26). First, muscle is a major consumer of resources (46), and, therefore, a competitor with the immune system for these resources. During an immune challenge in *Drosophila*, muscle reduces its own uptake of lipid (9) and glucose (26) due to increased activity of the Jak/Stat pathway. The result is an increase in energy availability for the immune system (26). The details of how the Jak/Stat pathway promotes the insulin resistance that helps reduce glucose uptake remains uncertain (9, 40). Nevertheless, these intra-muscular metabolic changes are vital for an effective cell-mediated immune response in *Drosophila* (26).

Secondly, muscle donates its own resources to the immune system during an immune response, as shown in *Drosophila* (i.e. glucose, 26, 47). Muscle is one of the largest sources of stored glycogen in the insect body (46), making it a vital source of fuel for the immune system. The liberation of glucose from muscle allows hemocytes to maximize their function against invaders (43). Without this release of glucose from muscle glycogen, cell mediated immunity is drastically reduced in larval *Drosophila* (47).

Thirdly, muscle helps mediate the necessary metabolic changes in other organs to ensure resource availability for the immune system. During an immune response, muscle releases signaling molecules (e.g. myokinins and glutamate) that help promote lipid mobilization in the fat body (35, 46). In *Drosophila*, muscle also redirects resources away from non-immune organs (9, 12), by secreting myokinins (e.g. myoglianin) and ImpL2 (26, 32). These chemical signals reduce the release of insulin-like peptides (ILPs) by the brain, leading to widespread reductions in glucose uptake in other tissues (26, 32). Muscle’s assistance in redirecting resources during an immune response is critical for effective cell-mediated immunity (26).

Many questions about immune-muscle connections remain to be resolved. For example, both McMullen et al. (26) working with larval *Drosophila*, and Yang et al. (48), working with adult *Drosophila*, find that elevated levels of the cytokine Upd3 reduces muscle-dependent behavior (i.e. locomotion in larvae, and climbing in adults). Based on their results, McMullen et al. (26) postulate that the decline in muscle activity reflects the decline in muscle glycogen and the lack of glucose uptake by muscle in response to raised levels of Upd3. However, Yang et al. (48), using a brain infection model that also results in increased Upd3 levels systemically, argue that the decline in muscle ability is unrelated to glucose. Adult fly muscle showed increased glucose content during an immune response in their study, and an increase in glucose uptake (48), although the data for that seems to be the increase in muscle glucose, as opposed to a direct measure of uptake. Yang et al. (48) suggest that the decline in muscle capacity during an immune response is due to changes in muscle mitochondrial metabolism, and/or neurodegeneration of motorneurons due to immunopathology (48, Supplementary Figure S1L). More research is needed to resolve this discrepancy. The lack of concordance between the two studies may reflect a difference between the response of adult and larval *Drosophila* muscle during an infection. It may also be due to differences between an immune response to a localized brain infection versus a parasitoid egg in the abdomen. In larvae, it is clear that without additional glucose from muscle, cell-mediated immunity is impaired (26). However, it is unclear what function muscle plays during a localized brain infection (48). There are also many methodological differences between the two studies, making direct comparison difficult. For example, the two studies use very different time points, i.e. less than 24 h after infection (26), compared to days after infection (48). The two papers may be demonstrating the progression of muscle’s immune response over time. Finally, both studies were done in *Drosophila*. Little is known about the relationship between muscle and the immune system in other insects. A wider phylogenetic perspective would help put these results in context, as discussed below.

## 4 Changes during an immune response may reflect both the shifting of resources, as well as mechanisms to compensate for muscle resource loss

Muscle, with its abundant glucose resources (stored as glycogen), is an obvious source of energy for the immune system. As described in the previous section, the liberation of glucose from muscle plays an important role in both humoral and cell-mediated immunity. However, the recruitment of muscle during an immune response is not without costs (e.g. *M. sexta*, 45, *Drosophila*, 26). Nevertheless, these negative impacts of immune activation are not necessarily a sign of immune dysregulation, i.e. a failure of the immune system’s regulatory network leading to damage (49). Instead, these changes may be a sign of a coordinated reconfiguration of physiological networks, optimizing the organism to deal with the present crisis. For example, during an immune response, glucose uptake in muscle in response to ILPs is reduced (26). This insulin resistance can cause muscle wasting, and has been considered a pathological condition in *Drosophila* (50). However, insulin resistance is also the mechanism by which the immune system is given priority for energy resources, required for an effective immune defense (26). In other words, insulin resistance is one of the mechanisms that allows the immune system to be fully effective. Therefore, insulin resistance in muscle during inflammation may not always be a sign of dysregulation, but may be part of the reconfiguration of physiological networks needed in order to support immunity.

The loss of muscle capacity has serious implications for behaviors that require intense muscle activity (e.g. anti-predator activity). For example, during an immune response, *M. sexta* caterpillars show a decline in muscle glycogen content, a reduction of the force of its anti-predator defensive strike, a reduced ability to evade parasitic wasps, and an increased risk of death (45). Larval damselflies (*Coenagrion puella*) exposed to bacteria have lower abdominal muscle mass, and a reduction in their swimming speed, suggesting that their anti-predator behavior is compromised (51). A number of species across phyla show a reduction in the effectiveness of anti-predator behavior during infection (e.g. crickets, 52; vertebrates, 53). Despite the serious costs related to muscle’s involvement in the immune system, these costs are not necessarily a sign of immune dysregulation. Because selection favors fitness, if reducing muscle capacity to boost immunity usually results in greater reproductive success, then this strategy will spread in the population.

However, selection will also favor animals with counter-strategies that limit the costs of muscle’s involvement in the immune response. One solution, often considered a ‘pathology’ in the past, is sickness behavior (54). Sickness behavior is the collection of behaviors animals adopt during an immune challenge that reduces the cost of infection, and promotes the reallocation of resources to the immune system (insects: 55, mammals: 56). For example, some sickness behaviors reduce the risk of predation by increasing shelter use. This behavioral change results in a decrease in predator encounters, reducing the cost of muscle weakness (mammals, 54, insects, 55; fish, 57). Sickness behavior also includes the reduction of other muscle-demanding behaviors. For example, immune-challenged male crickets (*Gryllus texensis*) are less likely to fight, even in the presence of females (58). It has been hypothesized that reducing the need for fight-or-flight behavior allows more resources to be devoted to overcoming infection (54). However, it has remained unclear how and why this occurs. The recent research demonstrating trade-offs between the immune system and muscle (e.g. 26) helps explain why there is a behavioral shift during infection in many animals. If connections between the immune system and muscle cannot be uncoupled because of the energy demands of the immune response, then it would be expected that many species will alter their behavior during infection. Given the reduction in muscle capacity, reducing behaviors that require intense activity would reduce the animal’s risk of damage and death. Even if these changes in behavior seem to be negative (e.g. malaise) they are not necessarily a sign of immune dysregulation, but may be an evolved strategy to reduce the risk of death due to the effects of the immune response on muscle.

Many insects require bouts of muscle activity that cannot be easily reduced due to time constraints (e.g. due to limited mating opportunities). Therefore, it is perhaps unsurprising that an immune challenge does not always reduce courtship behavior in insects (4). For example, *Drosophila* males continue to court females even while mounting an immune response (59). Courting males require intense effort from specific muscles in order to produce their courtship song (60). Therefore, immune activation spares at least some muscles in *Drosophila* males during courtship. The relationship between muscle and the immune system should be examined under courtship conditions in *Drosophila*. Such a study could illuminate novel methods of boosting immune function without impacting muscle.

Muscle’s importance for redirecting energy resources allows it to participate in the redirection of resources between the immune system and other organ systems. For example, muscle plays a role in the physiological trade-offs between reproduction and immunity in female *Drosophila* (4) by regulating lipid mobilization during infection (35). During aging, many animals increase their investment in reproduction (61). This phenomenon, called terminal reproductive investment, can provide animals with a fitness advantage despite the concomitant immunosuppression (61). Muscle may facilitate terminal reproductive investment by altering resource flow within the organism. Muscle may play an important role in producing adaptive shifts in immune function.

### 4.1 Both pathogens and the immune response can damage muscle directly

In addition to muscle losing resources during an immune response, both pathogens and the immune response can damage muscle. In larval *Drosophila*, exposure of skeletal muscle to lipopolysaccharides, a pathogen associated molecular pattern (PAMP), leads to muscle cell hyperpolarization and a reduction in the amplitude of excitatory junction potentials (EJPs) (62). Reduced EJPs means less excitation and activation of muscle (63). PAMPs like lipopolysaccharides appear to have direct effects on ion channels in both muscle and motor neurons (64). PAMPs can also interrupt signaling systems between muscle and other organs. LPS appears to block muscle glutamate receptors (65). Furthermore, muscle is susceptible to oxidative damage generated by the immune system (i.e. immunopathology). For example, the increase in phenoloxidase activity in larval damselflies (*C. puella*) exposed to bacteria leads to oxidative damage in muscle and reduced swimming speed (51, 66). Therefore, a decline in muscle function during an immune response can be the result of: a shift in resources away from muscle, damage by the pathogen (a cost of infection), and/or damage by the immune system (a cost of immune defense). Discovering the exact causes of immune-related declines in muscle capacity will require a detailed understanding of the cellular and molecular mechanisms leading to reduced muscle function. Such studies will also require manipulations of the system (e.g. effects of immune activation on muscle with and without the pathogen).

### 4.2 Chronic inflammation as an adaptive strategy

Clinical immunology divides inflammation into two types: acute and chronic (67). Acute inflammation encompasses the immediate response of the immune system to a pathogen (67). Although it can be damaging, it exists for a brief period and subsides as infection wanes (67). Chronic inflammation, on the other hand, is frequently induced by non-pathogenic events, is long-lasting, and appears to continue without any apparent purpose or resolution (67). Because chronic inflammation is damaging, it has been characterized as immune dysregulation (49). The insect immune system can exhibit this type of dysregulation too. Insects also show chronic inflammation, i.e. increased immune activity that is long-lasting leading to organ damage. For example, older adult beetles (*Tenebrio molitor*) exhibited more Malpighian tubule damage after an immune challenge than younger beetles. This increased damage was caused by the increased phenoloxidase activity observed in older animals (6). Older beetles had chronically elevated levels of phenoloxidase activity compared with that of younger beetles, even without an immune challenge (6). Khan et al. (6) suggest that the immune system becomes dysregulated with age in these beetles. However, is chronic inflammation (i.e. chronically elevated immune function) always a sign of dysregulation? Below are examples in which chronic inflammation may be adaptive in insects, despite it destructive effects on muscle and other organs.

A number of non-pathogenic events lead to chronic immune activation in insects. In *Drosophila*, starvation results in increased AMP production via a non-Toll receptor pathway (68). Food limitation (69), and changes in nutrition (70), can lead to increased phenoloxidase activity lasting days, a sign of chronic inflammation (6). In *M. sexta* caterpillars, chronic immune activation due to fasting occurs because of a reconfiguration of the immune system. During immune system reconfiguration, the dynamics and amplitude of different immune components are altered. For example, fasting in *M. sexta* produces an upregulation of constitutive immunity, with some components, such as AMPs, switching from inducible to constitutive transcription (69). There is also an upregulation of phenoloxidase activity due to a rewiring of this biochemical pathway (69). The expression of inhibitors of the phenoloxidase pathway, such as *serpin 3*, are down-regulated and expression of activators such a *phenoloxidase activating enzyme 3* are upregulated (69). These changes lead to increased phenoloxidase activity in fasting caterpillars, even in the absence of an immune challenge (69). Phenoloxidase activity produces reactive molecules (e.g. ROS) (16) that can destroy pathogens (71). However, it also damages muscle and other tissues (6, 66). The chronic increase in phenoloxidase activity that occurs during fasting may help to compensate for reductions in other immune components (69). Some immune components, such as hemocytes, perform poorly when resources are short (e.g. in *Drosophila*, 26). Therefore, chronic inflammation during starvation may be a sign of a change in the immune response to compensate for the decline of some immune components.

Chronic inflammation could also be adaptive in environments with high pathogen prevalence. Populations of *Drosophila* exposed to high rates of parasitic wasps show increased hemocyte numbers, increasing the ability of larval *Drosophila* to encapsulate parasitic wasp eggs (72). The cytokine Upd3 is chronically increased, as even immature hemocytes (i.e. lamellocytes) secrete it (73). This increased secretion of Upd3 leads to chronically increased Jak/Stat activity in muscle (73), leading to an enhancement of both humoral and cell-mediated immunity (73). Although the duration of this effect is unclear, under abundant food conditions it appears to last as long as the stimulus is present (73). This chronic increase in Upd3 has negative consequences for muscle, such as a loss of muscle mass (50). Nevertheless, it provides larval *Drosophila* with stronger, faster immune responses, giving them a better chance of survival in an environment with high parasitism rates (73). Similarly, after clearing an infection, some insect species show a long-lasting increase in immune activity. For example, immune-challenged bumble bees (*Bombus terrestris*) show an increase in lysozyme-like activity that lasts at least 2 weeks after the initial challenge (74). Such an increase in immune activity improves the response to subsequent infections (14). Despite the damage caused by chronic up-regulation of immune responses, under pathogen-filled conditions such chronic inflammation may be adaptive.

In mammals, chronic elevation of immune activity leads to a loss of energy reserves, and other negative effects, and it is considered pathological (75). However, animals showing chronic inflammation are usually compared to animals in optimal situations, i.e. abundant food and low infection risk. Under these conditions, chronic immune activity, whether it be in mammals or insects, will always appear maladaptive due to its costs. However, animals showing chronic inflammation should be compared to animals in which food availability is low and/or infection prevalence is high. Preventing animals from producing chronic inflammation (e.g. by using RNAi to suppress cytokine production) under suboptimal conditions would also help demonstrate potential positive effects. Chronic inflammation may provide benefits that exist only under adverse conditions, and when viewed on the level of the whole organism (i.e. effects on fitness). The insect examples show that chronic inflammation may sometimes be an adaptive solution when the environment is suboptimal, even if it leads to damage in tissues such as muscle. In other words, some forms of chronic inflammation may be an evolved strategy that optimizes the immune response to maximize fitness when conditions are suboptimal (76).

## 5 Conclusions

A strong immune response is required only intermittently in the life of an insect. The sporadic nature of pathogen exposure has probably contributed to the evolution of the immune system as a complex, multi-organ network. By calling upon the resources of other organ systems during an immune response, fewer resources are tied up unnecessarily in the immune system. In insects, muscle is an active participant in the immune response. Muscle produces antimicrobial molecules, provides needed energy resources, and helps rebalance metabolic pathways to redirect resources to immune cells (**Figure 1**). By recruiting additional tissues like muscle, the immune system enhances its response to life-threatening infections.

Unfortunately, our understanding of these interactions is incomplete, and almost non-existent for insects other than *Drosophila*. However, immune-muscle interactions have been documented in other insects (e.g. lepidopterans) suggesting that these interactions are not restricted to *Drosophila*. Given the selective forces shaping the interactions between immune function and muscle, examining muscle’s involvement in different insect species, under different conditions (e.g. during food limitation, courtship, and different levels of pathogen and predator prevalence) would help us understand some of the evolutionary forces shaping immune function. How immune system networks adapt to different conditions is important information given the growing environmental challenges insects face.

Finally, viewing the immune system as an organism-wide response suggests that some examples of immune dysregulation may be due to the reconfiguration of physiological networks in the face of unfavorable conditions. For example, chronically high levels of phenoloxidase activity, although damaging to muscle, may compensate for declines in other immune components when resources are scarce. Chronic inflammation may allow an animal to produce the best immune response possible given its circumstances. In other words, some types of chronic inflammation may be a feature of the interconnected immune system, not a bug.
